# Symptom clusters for revising scale membership in the analysis of prostate cancer patient reported outcome measures: a secondary data analysis of the Medical Research Council RT01 trial (ISCRTN47772397)

**DOI:** 10.1007/s11136-017-1548-y

**Published:** 2017-03-28

**Authors:** Agnieszka Lemanska, Tao Chen, David. P. Dearnaley, Rajesh Jena, Matthew. R. Sydes, Sara Faithfull

**Affiliations:** 1 Faculty of Health and Medical Sciences, School of Health Sciences, University of Surrey, Guildford, UK; 2 Department of Chemical and Process Engineering, University of Surrey, Guildford, UK; 3 The Institute of Cancer Research & Royal Marsden NHS Foundation Trust, London, UK; 4 University of Cambridge Department of Oncology, Cambridge Biomedical Campus, Addenbrooke’s Hospital, Cambridge, UK; 5 MRC Clinical Trials Unit at UCL, Institute of Clinical Trials and Methodology, London, UK

**Keywords:** Symptom clusters, UCLA-PCI, Urinary symptoms, PROMs analysis, Summative scores

## Abstract

**Purpose:**

To investigate the role of symptom clusters in the analysis and utilisation of patient reported outcome measures (PROMs) for data modelling and clinical practice. To compare symptom clusters with scales, and to explore their value in PROMs interpretation and symptom management.

**Methods:**

A dataset called RT01 (ISCRTN47772397) of 843 prostate cancer patients was used. PROMs were reported with the University of California, Los Angeles Prostate Cancer Index (UCLA-PCI). Symptom clusters were explored with hierarchical cluster analysis (HCA) and average linkage method (correlation > 0.6). The reliability of the Urinary Function Scale was evaluated with Cronbach’s Alpha. The strength of the relationship between the items was investigated with Spearman’s correlation. Predictive accuracy of the clusters was compared to the scales by receiver operating characteristic (ROC) analysis. Presence of urinary symptoms at 3 years measured with the late effects on normal tissue: subjective, objective, management tool (LENT/SOM) was an endpoint.

**Results:**

Two symptom clusters were identified (urinary cluster and sexual cluster). The grouping of symptom clusters was different than UCLA-PCI Scales. Two items of the urinary function scales (“number of pads” and “urinary leak interfering with sex”) were excluded from the urinary cluster. The correlation with the other items in the scale ranged from 0.20 to 0.21 and 0.31 to 0.39, respectively. Cronbach’s Alpha showed low correlation of those items with the Urinary Function Scale (0.14–0.36 and 0.33–0.44, respectively). All urinary function scale items were subject to a ceiling effect. Clusters had better predictive accuracy, AUC = 0.70 –0.65, while scales AUC = 0.67–0.61.

**Conclusion:**

This study adds to the knowledge on how cluster analysis can be applied for the interpretation and utilisation of PROMs. We conclude that multiple-item scales should be evaluated and that symptom clusters provide a study-specific approach for modelling and interpretation of PROMs.

## Introduction

Patient reported outcome measures (PROMs) are health questionnaires that are completed directly by patients to measure patients’ health status or health-related quality of life (HRQOL). In radiotherapy, PROMs are used to obtain an insight into patients’ perceptions of the impact of their cancer and the consequences of treatment [1]. The advantage of PROMs over other patient monitoring techniques is that they provide information as perceived by patients. Therefore, PROMs add an important dimension to the information gathered by professional assessments or clinical tests. The patient’s perspective provides a holistic and a more comprehensive assessment of the treatment and PROMs are increasingly being seen as a way to improve practice by enhancing communication, improving management of symptoms associated with cancer or treatment as well as identifying patient care needs.

With significant improvements in rates of long-term survival [2], longitudinal PROMs in prostate cancer play a particularly important role as they offer the ability to assess and address health concerns or HRQOL issues of individual patients [3–5]. Other important clinical applications of PROMs include aiding treatment choices as well as identifying high-risk cancer patients, to achieve the best possible long-term health-related outcomes [6, 7]. These are all key challenges of modern oncology and PROMs play a strategic role in this as they enable tailored treatments and outcomes according to priorities, risks or concerns of individual patients [8, 9]. However, the successful application of PROMs in this area requires a deeper understanding of the methods for extracting information carried within PROMs [1]. PROMs data are complex, with large number of variables (HRQOL, symptoms, function, bother, performance or heath concerns) measured on different scales (with different levels, ratios or frequencies) and with confounders that can be attributed to cancer treatment or individual patient characteristics.

To extract and interpret information contained within PROMs summative scores are used (individual items are usually pooled together using average scores, or less commonly using the highest score). In tools such as the University of California, Los Angeles Prostate Cancer Index (UCLA-PCI), items are arranged in scales according to the underlying function or health concern. These scales are developed and carefully validated using clinical data [10, 11], and they are used to create agglomerate scores [11, 12]. However, this generic approach of summarising PROMs may become less sensitive or less specific for new treatments or changing patient populations. It has been shown that different treatments or patient populations (with different recruitment factors such as age or baseline characteristics) can generate different symptom profiles or symptom prevalence [13–16]. An alternative method of grouping symptoms for the purpose of extracting meaningful information and utilisation of PROMs in data modelling or clinical decision making is to use symptom clusters. Symptom clusters are groups of 3 or more correlated symptoms that occur together, and this is stable over time [17, 18]. The advantage of exploring symptom clusters within a dataset is that it allows a study-specific method of grouping symptoms, as symptom clusters can be easily determined specifically to each dataset or clinical trial. With that symptom, clusters have the potential to improve sensitivity and specificity to symptom grouping. Only items that are strongly correlated, and so measure the same underlying health concern, are included in summative scores.

The concept of symptom clusters is well known, and the methodology to define symptom clusters within PROMs data is well established [19–21]. The idea behind utilising symptom clusters in data modelling or symptom management is that symptoms in clusters are concurrent, and exert influence on one another. Symptom clusters were shown to be beneficial in providing effective symptom management in chemotherapy cancer patients (with various cancers) [22], and for determining multiple-symptom management strategies in cancer nursing [23]. It has been shown in HRQOL studies that managing symptoms in isolation is not as effective as managing symptoms in clusters [24–27]. This may be explained by common mechanisms of occurrence or common aetiology of symptoms in clusters [18, 28]. However, the relationship between symptoms in clusters is not yet fully understood.

We hypothesised that the study-specific grouping of PROMs items using symptom clusters provides a more sensitive and specific approach for the analysis of PROMs than the generic tool’s scales. Symptom clusters have the potential to identify patients at high risk of symptoms or side effects more accurately. This is because they allow for the grouping of items and calculating composite scores from items that are highly correlated, so they exhibit similar prevalence and are present in the same patients. In contrast, scales group symptoms according to the predefined disease and treatment-specific function or bother. This is important because of the possibility that trends or prevalence of symptoms for the same scale may vary from study to study, for example, according to the treatment modality or patient population. Radiotherapy prostate cancer patients experience different symptoms than those that have undergone prostatectomy [16, 28]. With the generic approach using scales for summarising PROMs, items that have different prevalence or trends in a study population will be included in the average score [29]. This may result in the loss of sensitivity in identifying patients at high risk of symptoms, or in alerts being created for patients with high symptoms that are not related to the same underlying health concern leading to loss of specificity. Therefore, symptom clusters have the potential to allow more meaningful PROMs data analysis and interpretation as symptom grouping can be calculated in a way that is unique to each study.

## Materials and methods

### Dataset and PROMs tool

In this study, we used MRC RT01 (ISCRTN47772397), a dataset consisting of 843 prostate cancer patients in a randomised controlled trial coordinated for the UK Medical Research Council (MRC) [30, 31]. MRC RT01 was a large, multicentre UK trial of patients with localised prostate cancer. Patients were randomly assigned to standard-dose (64 Gy) or escalated-dose (74 Gy) conformal radiotherapy (CFRT), which were both administered with neoadjuvant androgen suppression [32–35]. PROMs data were collected longitudinally with the UCLA-PCI. Data were gathered from the study cohort at ten time points (pre-hormone therapy, pre-radiotherapy, week 10, month 6, 12, 18, year 2, 3, 4 and 5 after the start of radiotherapy). The numbers of completed UCLA-PCI questionnaires at each point in time were 578, 757, 738, 712, 689, 655, 645, 594, 515 and 425, respectively. UCLA-PCI includes 20 items measuring six scales of function and bother in the three primary prostate cancer concern areas (urinary, bowel and sexual) [10]. To demonstrate the concepts presented in this study, we focused on the urinary domain of health (presented in the Appendix, Fig. 4). There are two scales (function and bother) in UCLA-PCI urinary domain. These are a five-item urinary function scale (“urinary leak”, “urinary control”, “dripping/wetting”, “number of pads” and “urinary leak interfering with sex”) and a single-item urinary bother scale (“How big a problem has urinary function been”; referred throughout as “urinary bother”).

The endpoint for statistical modelling and prediction was the measurement of late urinary symptoms at year 3 using the physician completed grading tool: late effects on normal tissue: subjective, objective, management tool (LENT/SOM) [36]. The aim of the analysis was to compare the predictive power of the urinary cluster to the urinary function scale. Literature has shown that early symptoms are often a precursor of late symptoms [37]. A binary variable of LENT/SOM recording any bladder or urethra symptoms (yes or no) was used. Data at year 3 were available for 725 patients. Data from year 3 post-radiotherapy were used because this post-treatment period has been shown to be representative of late urinary symptoms [30, 38]. While early side effects develop during radiotherapy and usually improve within the first few months after treatment, late side effects emerge months after radiotherapy, continue to develop post-treatment [39, 40] and usually get worse over subsequent years [38, 41]. Year 3 was used rather than years 4 or 5 due to the increasing number of drop-outs after 3 years. In year 4, the number of completed LENT/SOM questionnaires decreased to 621 and again in year 5 to 521.

### Data pre-treatment and missing data

Data analysis was performed at each time point for patients that completed their UCLA-PCI questionnaires in the respective time window. Intermittent missing data were treated with multiple imputation using seven imputations. Multiple imputation was used rather than complete case analysis in order to minimise the risk of biased results and to preserve sample size [42–44]. Although five imputations are recommended in theory as being sufficient [45], seven imputed datasets were created to further reduce uncertainty in the prediction of missing values from the imputation process [46, 47]. Clustering analysis was also performed on complete cases and the results were identical. The results of exploratory data analysis looking at prevalence of urinary domain items were shown for complete cases. The number of missing data is also indicated. In addition to treating missing data, variables were also rescaled to correct for the difference in scales. Variables were recorded on a Likert scale with levels from 3 to 6 so they were rescaled from 0 to 100 as recommended by the scoring manual [12] so that they have the same impact on the analysis. Variables that were negatively worded were reversed.

### Symptom clustering

Clustering of patient-reported symptoms from the UCLA-PCI was performed at ten points in time. The similarity between symptoms was measured with Spearman’s rho correlation coefficient (*r*_*s*_) [48]. To obtain pooled results from seven multiple imputed datasets, composite correlation matrices were calculated for each time point using Fisher’s z transformation [49, 50]. Clustering between the symptoms was identified using hierarchical cluster analysis (HCA) with average linkage method of cluster agglomeration. Symptom clusters were determined at a cut-off correlation value of >0.6 [19, 20].

### Strength of the association between symptoms in the urinary function scale

To measure the reliability of the UCLA-PCI urinary function scale, Cronbach’s alpha was used [51]. This is a statistic that estimates scale reliability based on correlation between items. It is the most commonly reported reliability estimate in healthcare for multiple-item scales [52]. It was also used to test the reliability of the UCLA-PCI during tool development [10] and therefore it is reported here. It is important to acknowledge that Cronbach’s alpha usually underestimates the reliability (provides a very conservative lower bound of the estimate). Other statistics such as Lambda2 [53] or greatest lower bound (glb) [54] are available and provide more accurate estimate (higher value of the lower bound) of the true reliability [55, 56]. In addition, Cronbach’s alpha does not reflect the complexity (in relation to factorial dimensionality) of the scale, or in other words, whether the items in the scale measure one or more related constructs. Factor analysis can be used to explore this [21]. Cronbach’s alpha also does not inform whether the scale measures the construct that it was developed to measure, e.g. whether the urinary function scale measures urinary function or another health-related issue. However, because testing the dimensionality or validity of the urinary function scale was not the aim of these analyses, we report Cronbach’s alpha with alpha’s standard error (ASE) [57] to explore the reliability of the scale in the MRC RT01 dataset and to analyse the contribution of the five items in assessing the underlying health concern (urinary function). This is to investigate whether scale reliability is a sample specific concept and whether the reliability estimate measured with statistics such as Cronbach’s alpha reflects a general reliability of the tool or rather the reliability in a specific population. Alpha ≥ 0.7 was used as a cut-off value for the acceptable reliability of a scale [58]. Each item was tested by calculating the scale alpha without the tested item, and by calculating the correlation of each item with the scale (not corrected and corrected for the item). This was done to measure the relatedness of each item with the scale and to explore whether the item should be included in the scale in the MRC RT01 dataset.

### Predictive value of early urinary cluster and early urinary function scale

The power to predict late urinary symptoms by the early clusters (data were summarised according to clusters) was tested and compared to the scales (data were summarised according to item grouping outlined by the original scales). The analysis was performed with the receiver operating characteristic (ROC) curve analysis for six points in time (pre-hormone therapy, pre-radiotherapy, week 10, month 6, 12 and 18). The specificity and sensitivity in predicting late urinary symptoms was calculated and models evaluated with the area under the curve (AUC) values. All the analyses were performed with R version 3.0.2 (R Foundation for Statistical Computing, Vienna, Austria).

## Results

### Prevalence of urinary symptoms

**Fig. 1:**
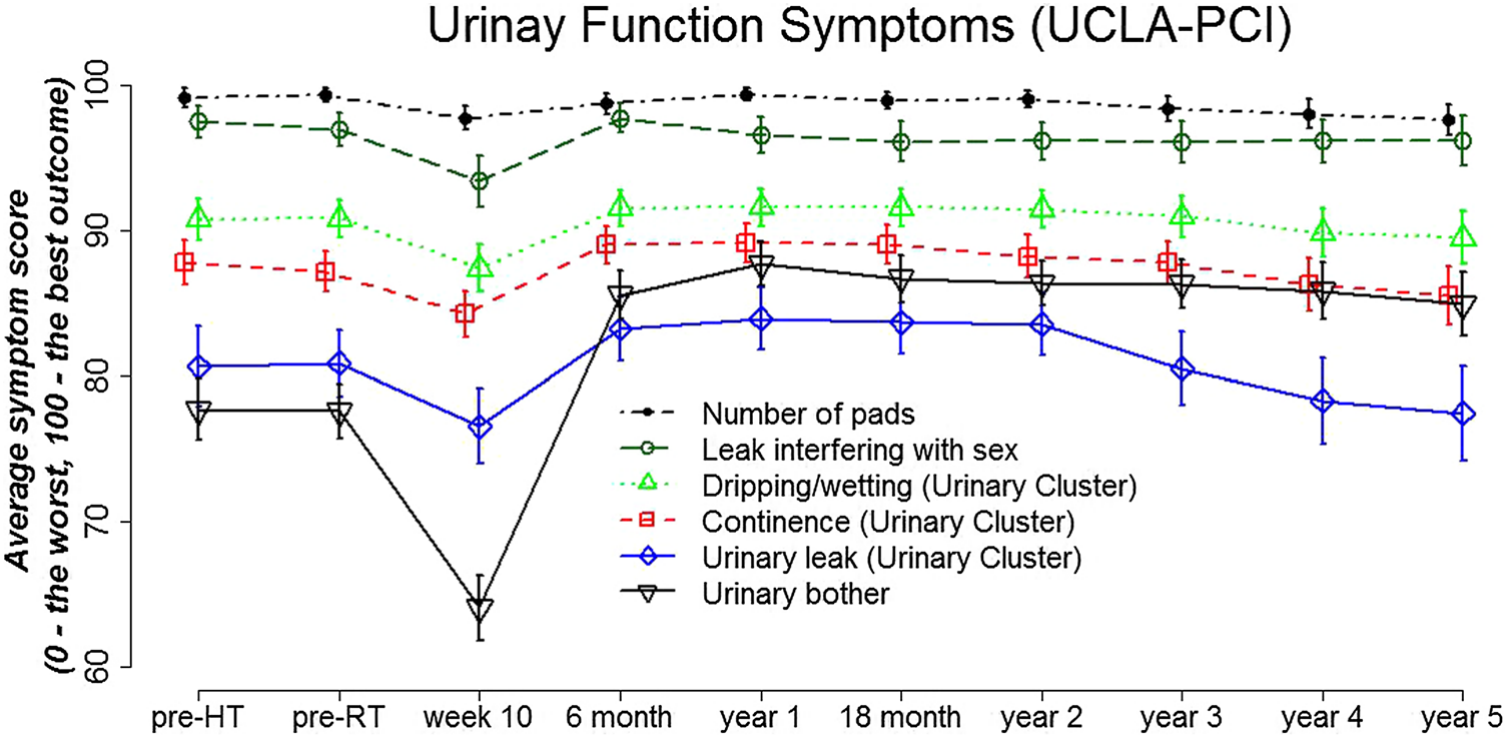
Longitudinal profiles of items in the University of California, Los Angeles Prostate Cancer Index (UCLA-PCI), Urinary Domain of Health

Baseline characteristics of the MRC RT01 study participants are presented in Table 1 and are reported in more detail elsewhere [30]. The time profiles of mean scores of the urinary function scale items with the 95% confidence intervals (Fig. 1) show that the prevalence of different urinary symptoms varied significantly over time. Items such as “Number of pads” and “Urinary leak interfering with sex” were rarely reported and average scores were close to 100. Only 1% (pre-radiotherapy and pre-hormone therapy) to 4% (week 10 and year 5) of patients reported using any pads, and only 4% (month 6) to 8% (week 10) reported “Urinary leak interfering with sex” (Table 2). The remaining three symptoms of the Urinary Function Scale were significantly more prevalent (Fig. 1). Some level (score < 100) of “Urinary leak”, “Urinary control” and “Dripping/wetting” was reported on average by 32% (28.3% year 1 to 36.0% year 5), 33% (29.0% year 1 to 39.8% week 10) and 27% of patients (23.7% year 1 to 31.5% week 10), respectively (Table 2). From those three symptoms, the mean values of “urinary leak” were the lowest over time (76.6 week 10 to 80.9 pre-radiotherapy), showing this symptom had the worst outcome. More patients reported “urinary bother” (70.6% week 10 to 33.6% year 1) than any other symptom in the Domain. This symptom also had the worst mean scores before and during treatment (Fig. 1). It improved by month 6 and remained higher than baseline levels throughout follow-up.

**Table 1:** Patient baseline characteristics

Characteristic	
Treatment, *n* (%)	
Standard	421 (50)
Escalated	422 (50)
Age (years)	
Mean (SD)	67.1 (6.0)
Stage, *n* (%)	
T1	209 (25)
T2	475 (56)
T3	147 (17)
Missing	12 (1)
Gleason score, *n* (%)	
2–4	70 (8)
5–6	411 (49)
7	191 (23)
8–10	96 (11)
Missing	75 (9)
Pre-hormone PSA (ng/ml)	
Mean (SD)	15.4 (9.8)
Missing	6 (1)
Pre-existing co-morbidities, *n* (%)	
Diabetes	55 (6)
Hypertension	252 (30)
Inflammatory bowel or diverticular disease	36 (4)
Haemorrhoids in past 12 months	89 (11)
Previous pelvic surgery, *n* (%)	48 (6)
Previous TURP, *n* (%)	100 (12)

**Table 2:** Prevalence of Urinary Domain items of the University of California, Los Angeles Prostate Cancer Index (UCLA-PCI)

Point in time and score	Ur. leak	Ur. contr	Drip/wet	Pad no.	Interf. sex	Ur. bother
Pre-hormone therapy (*N* = 578)						
Mean (SD)	80.7 (33.3)	87.8 (18.6)	90.8 (17.3)	99.2 (8.1)	97.5 (12.8)	77.7 (25.6)
100—no symptom, *n* (%)	403 (69.7)	382 (66.1)	413 (71.5)	566 (97.9)	504 (87.2)	265 (45.8)
>0 and <100, *n* (%)	117 (20.2)	188 (32.5)	155 (26.8)	3 (0.5)	20 (3.5)	303 (52.5)
0—worst symptom, *n* (%)	54 (9.3)	3 (0.5)	2 (0.3)	3 (0.5)	5 (0.9)	8 (1.4)
Missing, *n* (%)	4 (0.7)	5 (0.9)	8 (1.4)	6 (1.0)	49 (8.5)	2 (0.3)
Pre-radiotherapy (*N* = 757)						
Mean (SD)	80.9 (32.2)	87.2 (19.1)	90.8 (17.4)	99.4 (6.6)	97.0 (14.6)	77.6 (26.2)
100—no symptom, *n* (%)	512 (67.6)	484 (63.9)	537 (70.9)	737 (97.4)	628 (83.0)	350 (46.2)
>0 and <100, *n* (%)	172 (22.7)	254 (33.5)	199 (26.3)	5 (0.7)	26 (3.4)	385 (50.8)
0—worst symptom, *n* (%)	63 (8.3)	8 (1.1)	3 (0.4)	2 (0.3)	10 (1.3)	13 (1.7)
Missing, *n* (%)	10 (1.3)	11 (1.5)	18 (2.4)	13 (1.7)	93 (12.3)	9 (1.2)
Week 10 (*N* = 738)						
Mean (SD)	76.6 (35.2)	84.3 (21.4)	87.4 (22.0)	97.8 (11.0)	93.4 (22.0)	64.1 (30.4)
100—no symptom, *n* (%)	458 (62.1)	429 (58.1)	487 (66.0)	686 (93.0)	547 (74.1)	203 (27.5)
>0 and <100, *n* (%)	183 (24.8)	283 (38.4)	224 (30.4)	28 (3.8)	40 (5.5)	482 (65.3)
0—worst symptom, *n* (%)	81 (11.0)	10 (1.4)	8 (1.1)	2 (0.3)	20 (2.7)	39 (5.3)
Missing, n (%)	16 (2.2)	16 (2.2)	19 (2.6)	22 (3.0)	131 (17.8)	14 (1.9)
Month 6 (*N* = 712)						
Mean (SD)	83.2 (29.4)	89.0 (17.0)	91.6 (16.7)	98.7 (9.1)	97.8 (12.5)	85.6 (21.8)
100—no symptom, *n* (%)	484 (68.0)	472 (66.3)	508 (71.3)	665 (93.4)	579 (81.3)	420 (59.0)
>0 and <100, *n* (%)	165 (23.2)	214 (30.1)	171 (24.0)	11 (1.5)	19 (2.7)	260 (36.5)
0—worst symptom, *n* (%)	37 (5.2)	1 (0.1)	2 (0.3)	3 (0.4)	7 (1.0)	8 (1.1)
Missing, *n* (%)	26 (3.7)	25 (3.5)	31 (4.3)	33 (4.6)	107 (15.0)	24 (3.4)
Year 1 (*N* = 689)						
Mean (SD)	83.9 (28.6)	89.2 (18.0)	91.6 (17.0)	99.4 (5.4)	96.6 (15.7)	87.7 (20.1)
100—no symptom, *n* (%)	479 (69.5)	475 (68.9)	509 (73.9)	660 (95.8)	574 (83.3)	446 (64.7)
>0 and <100, *n* (%)	161 (23.4)	196 (28.4)	162 (23.6)	8 (1.2)	26 (3.8)	229 (33.3)
0—worst symptom, *n* (%)	34 (4.9)	4 (0.6)	1 (0.1)	0 (0.0)	12 (1.7)	2 (0.3)
Missing, *n* (%)	15 (2.2)	14 (2.0)	17 (2.5)	21 (3.0)	77 (11.2)	12 (1.7)
Month 18 (*N* = 655)						
Mean (SD)	83.7 (28.2)	89.0 (17.2)	91.6 (16.3)	99.0 (7.1)	96.2 (16.5)	86.7 (20.8)
100—no symptom, *n* (%)	446 (68.1)	440 (67.2)	473 (72.2)	625 (95.4)	516 (78.8)	405 (61.8)
>0 and <100, *n* (%)	165 (25.2)	197 (30.1)	163 (24.9)	13 (2.0)	27 (4.1)	229 (35.0)
0—worst symptom, *n* (%)	27 (4.1)	2 (0.3)	1 (0.2)	0 (0.0)	9 (1.4)	4 (0.6)
Missing, *n* (%)	17 (2.6)	16 (2.4)	18 (2.7)	17 (2.6)	103 (15.7)	17 (2.6)
Year 2 (*N* = 645)						
Mean (SD)	83.6 (27.6)	88.2 (18.7)	91.5 (16.5)	99.1 (7.2)	96.2 (15.4)	86.4 (19.5)
100—no symptom, *n* (%)	418 (64.8)	417 (64.7)	451 (69.9)	602 (93.3)	505 (78.3)	373 (57.8)
>0 and <100, *n* (%)	170 (26.4)	190 (29.4)	155 (24.0)	9 (1.4)	34 (5.3)	239 (37.1)
0—worst symptom, *n* (%)	26 (4.0)	4 (0.6)	2 (0.3)	1 (0.2)	8 (1.2)	2 (0.3)
Missing, *n* (%)	31 (4.8)	34 (5.3)	37 (5.7)	33 (5.1)	98 (15.2)	31 (4.8)
Year 3 (*N* = 594)						
Mean (SD)	80.5 (30.8)	87.8 (17.8)	91.0 (17.3)	98.4 (10.6)	96.1 (16.3)	86.3 (20.4)
100—no symptom, *n* (%)	374 (63.0)	373 (62.8)	410 (69.0)	552 (92.9)	474 (79.8)	353 (59.4)
>0 and <100, *n* (%)	160 (26.9)	194 (32.7)	150 (25.2)	10 (1.7)	27 (4.5)	216 (36.3)
0—worst symptom, *n* (%)	37 (6.2)	2 (0.3)	2 (0.3)	4 (0.7)	9 (1.5)	0 (0.0)
Missing, *n* (%)	23 (3.9)	25 (4.2)	32 (5.4)	28 (4.7)	84 (14.1)	25 (4.2)
Year 4 (*N* = 515)						
Mean (SD)	78.3 (33.0)	86.3 (20.0)	89.8 (18.6)	98.0 (11.2)	96.2 (16.1)	85.9 (21.6)
100—no symptom, *n* (%)	308 (59.8)	311 (60.4)	339 (65.8)	468 (90.9)	406 (78.8)	306 (59.4)
>0 and <100, *n* (%)	135 (26.2)	173 (33.6)	141 (27.4)	13 (2.5)	24 (4.7)	179 (34.7)
0—worst symptom, *n* (%)	45 (8.7)	4 (0.8)	5 (1.0)	3 (0.6)	8 (1.6)	3 (0.6)
Missing, *n* (%)	27 (5.2)	27 (5.2)	30 (5.8)	31 (6.0)	77 (15.0)	27 (5.2)
Year 5 (*N* = 425)						
Mean (SD)	77.5 (33.2)	85.5 (20.2)	89.5 (18.5)	97.6 (11.2)	96.2 (16.1)	84.9 (22.4)
100—no symptom, *n* (%)	252 (59.3)	247 (58.1)	279 (65.6)	386 (90.8)	331 (77.9)	243 (57.2)
>0 and <100, *n* (%)	118 (27.8)	151 (35.5)	122 (28.7)	17 (4.0)	19 (4.4)	157 (37.0)
0—worst symptom, *n* (%)	35 (8.2)	4 (0.9)	2 (0.5)	1 (0.2)	6 (1.4)	5 (1.2)
Missing, *n* (%)	20 (4.7)	23 (5.4)	22 (5.2)	21 (4.9)	69 (16.2)	20 (4.7)

*N* is the number of UCLA-PCI forms completed in each time point. Mean and standard deviation (SD) is calculated at each point in time for *N* patients

A ceiling effect (a large proportion (>15%) of patients reporting a maximum score [59–61]) was observed for all of the six items in the Domain. Symptoms were most prevalent in week 10 before the acute reaction to radiotherapy had settled. However, even in week 10, the percentage of patients reporting no symptoms (a score equal to 100) ranged from 27.5% for “urinary bother”, through to 58.1% for “urinary control” and up to even 93.0% for “number of pads”. Urinary symptoms were present in 39% of 725 patients at year 3 as reported by clinicians with LENT/SOM. This was comparable to urinary dysfunction reported by patients with UCLA-PCI. In year 3, 33.1, 33.0, 25.5 and 36.3% of patients reported experiencing “urinary leak”, “urinary control”, “dripping/wetting” and “urinary bother”, respectively. However, only 2.4 and 6.0% of patients reported using pads or “Urinary leak interfering with sex” in year 3.

### Symptom clusters

The analysis of symptom clusters identified 2 clusters with 3 or more symptoms (urinary and sexual). Bowel Cluster was not present. The symptom clusters were relatively stable over time, and core symptoms were present across time. However, cluster membership was different to that represented by the scales of the UCLA-PCI. The Urinary Cluster consisted of three symptoms (“urinary leak”, “urinary control” and “dripping/wetting”) out of the five urinary function scale items (see Appendix, Fig. 4). The remaining two symptoms (“number of pads” and “urinary leak interfering with sex”) did not exhibit high enough correlations to be included in the urinary cluster. The sexual cluster consisted of five core symptoms (“erection ability”, “orgasm ability”, “quality of erections”, “frequency of erections” and “overall sexual function”). Out of the remaining three items, two (“sexual desire” and “awoke with an erection”) were intermittently present in the sexual cluster over time, and one item (“Intercourse”) did not appear in the cluster at any point in time. The correlation of bowel symptoms in the bowel function scale was not strong enough to form a cluster at any point in time.

### Correlation and scale reliability

We analysed Spearman’s correlation of “number of pads” and “urinary leak interfering with sex” with the urinary cluster symptoms (Table 3). It emerged that the correlation of “number of pads” with the three urinary cluster symptoms ranged from 0.089 to 0.333, and the correlation of “urinary leak interfering with sex” ranged from 0.249 to 0.459. These low correlation values led to the exclusion of these items from the urinary cluster. Cronbach’s alpha was >0.7 for the urinary function scale at each point in time which indicated reliability of the scale. However, detailed analysis (Table 4) has shown that excluding the “number of pads” item would increase Cronbach’s alpha at all points in time. Dropping “urinary leak interfering with sex” from the scale did not decrease Cronbach’s alpha either (apart from pre-radiotherapy). It is also worth observing that excluding any of the urinary cluster symptoms from the urinary function scale led to a decrease in alpha below the acceptable value of 0.7 at all points in time apart from week 10. The correlation of “number of pads” and “urinary leak interfering with sex” with the urinary function scale showed (similarly to Spearman’s correlation analysis) low correlation which ranged from 0.143 to 0.358 for the “number of pads” and 0.330 to 0.439 for the “urinary leak interfering with sex”.

**Table 3:** correlation analysis for the items included in the Urinary Domain of health of the University of California, Los Angeles Prostate Cancer Index (UCLA-PCI)

	Urinary leak	Urinary control	Number of pads	Dripping/wetting	Urinary leak interfering with sex	Urinary bother
Urinary leak	1.00					
Urinary control	0.753 (0.700–0.809)	1.00				
Number of pads	0.214 (0.104–0.300)	0.202 (0.126–0.285)	1.00			
Dripping/wetting	0.698 (0.644–0.746)	0.694 (0.653–0.734)	0.209 (0.089–0.333)	1.00		
Urinary leak interfering with sex	0.326 (0.249–0.366)	0.306 (0.267–0.371)	0.200 (0.118–0.263)	0.389 (0.258–0.459)	1.00	
Urinary bother	0.505 (0.389–0.594)	0.549 (0.464–0.630)	0.168 (0.046–0.319)	0.527 (0.384–0.599)	0.271 (0.191–0.370)	1.00

Mean correlations over time calculated using Fisher Z transform. Ranges over time are shown in brackets

**Table 4:** Cronbach’s alpha reliability analysis to evaluate the internal structure of the Urinary Function Scale of the University of California, Los Angeles Prostate Cancer Index (UCLA-PCI). Alpha ≥ 0.7 was used as a cut-off for the acceptable internal consistency

Scale alpha = 0.721 ± 0.030	Alpha without the item	Correlation with the scale	Correlation with the scale without the item
Pre-hormone therapy			
Urinary leak	0.604 ± 0.038	0.800	0.688
Urinary control	0.614 ± 0.039	0.785	0.685
Number of pads	0.775 ± 0.032	0.474	0.205
Dripping/wetting	0.608 ± 0.039	0.795	0.698
Urinary leak interfering with sex	0.727 ± 0.033	0.584	0.330
Pre-radiotherapy			
Urinary leak	0.624 ± 0.032	0.790	0.683
Urinary control	0.625 ± 0.034	0.789	0.701
Number of pads	0.802 ± 0.027	0.426	0.143
Dripping/wetting	0.613 ± 0.033	0.807	0.709
Urinary leak interfering with sex	0.705 ± 0.029	0.651	0.414
Week 10			
Urinary leak	0.705 ± 0.031	0.826	0.717
Urinary control	0.724 ± 0.031	0.791	0.680
Number of pads	0.815 ± 0.026	0.583	0.358
Dripping/wetting	0.695 ± 0.032	0.844	0.750
Urinary leak interfering with sex	0.792 ± 0.027	0.642	0.439
Month 6			
Urinary leak	0.658 ± 0.033	0.806	0.710
Urinary control	0.670 ± 0.034	0.785	0.702
Number of pads	0.796 ± 0.027	0.520	0.253
Dripping/wetting	0.641 ± 0.034	0.833	0.744
Urinary leak interfering with sex	0.759 ± 0.028	0.610	0.365
Year 1			
Urinary leak	0.661 ± 0.033	0.829	0.739
Urinary control	0.681 ± 0.034	0.796	0.739
Number of pads	0.813 ± 0.027	0.507	0.255
Dripping/wetting	0.658 ± 0.033	0.835	0.738
Urinary leak interfering with sex	0.766 ± 0.028	0.624	0.386
Month 18			
Urinary leak	0.650 ± 0.035	0.832	0.714
Urinary control	0.677 ± 0.035	0.786	0.685
Number of pads	0.797 ± 0.029	0.532	0.296
Dripping/wetting	0.655 ± 0.035	0.824	0.712
Urinary leak interfering with sex	0.771 ± 0.030	0.596	0.380

Each item was tested by calculating (a) scale alpha ± alpha’s standard error (ASE) without the tested item (column 1), (b) not corrected correlation of an item with the scale (column 2) and (c) correlation of an item with the scale corrected for the item (column 3)

### Predicting late urinary symptoms (urinary cluster versus urinary function scale)

**Fig. 2:**
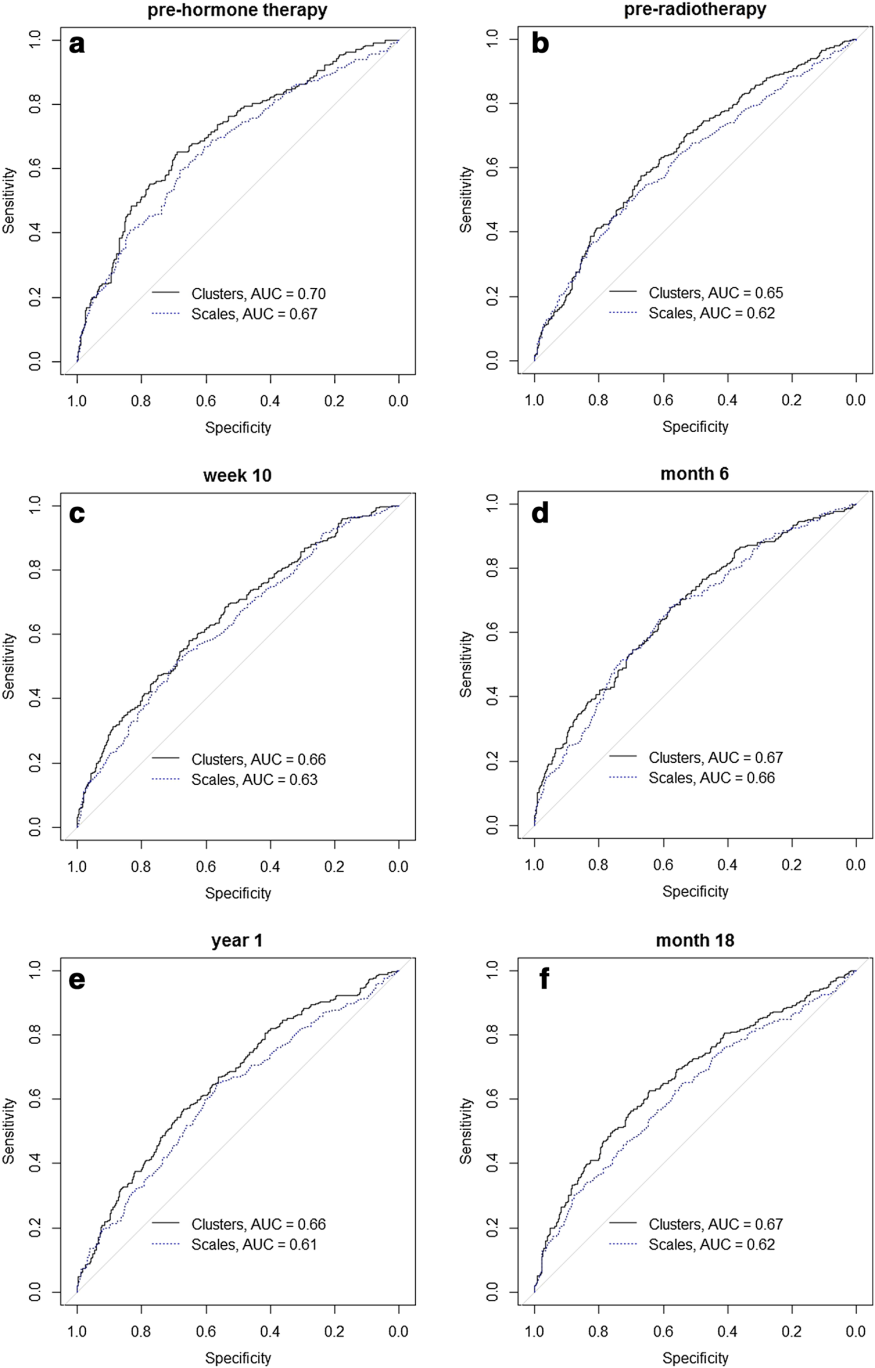
ROC curve analysis comparing predictive power of clusters to domains of the University of California, Los Angeles Prostate Cancer Index (UCLA-PCI). A binary item (yes/no) representing the presence or absence of urinary symptoms at year 3 post-radiotherapy was used as an endpoint. It was measured with the Late Effects on Normal Tissue: Subjective, Objective, Management tool (LENT/SOM), bladder/urethra section

The power of clusters to predict late urinary symptoms at year 3 was evaluated and compared to that obtained with UCLA-PCI scales. The results of ROC analysis are presented in Fig. 2. The best predictive accuracy, with the highest AUC = 0.70, was recorded for the baseline cluster data (pre-hormone therapy). In all time points, clusters showed better predictive power than the original UCLA-PCI scales. Although these differences were not found to be statistically significant, they were consistent over time.

### Discussion

When investigating patterns of urinary function, it was evident that men rarely reported using pads or “Urinary leak interfering with sex”. In fact, statistically significantly more men reported a certain degree of “Urinary leak” than using pads. This may be due to the difference in the scales that those items are recorded on. The frequency intervals that these items are recorded at may be incompatible. “Number of pads” is reported daily, while “urinary leak” is investigated weekly. The frequency for the other two items of the urinary cluster is not specified. “urinary control” is recorded as none, frequent or occasional and “Dripping/wetting” is recorded as a magnitude of a problem within the last 4 weeks (see Appendix, Fig. 4).

The key strength of this study is that MRC RT01 is a large dataset with a long follow-up of patients. The limitations of this study are the large number of drop-outs at each point in time and the fact that UCLA-PCI is a relatively old tool. However, the more recently developed expanded prostate cancer index composite (EPIC) instrument [11] (expanded from the UCLA-PCI) adapted similar scaling for its items so the findings of this study should still be applicable. Both UCLA-PCI and EPIC have been developed for general use in prostate cancer patients [62, 63]. However, it has been shown that different treatment modalities as well as different patient populations (differing recruitment factors such as age or baseline characteristics) generate different symptom profiles or symptom prevalence [13–16]. Therefore, with evolving treatments and changing characteristics of patient populations, study-specific approaches to analysing PROMs are warranted and symptom clusters can be used for this purpose.

**Fig. 3:**
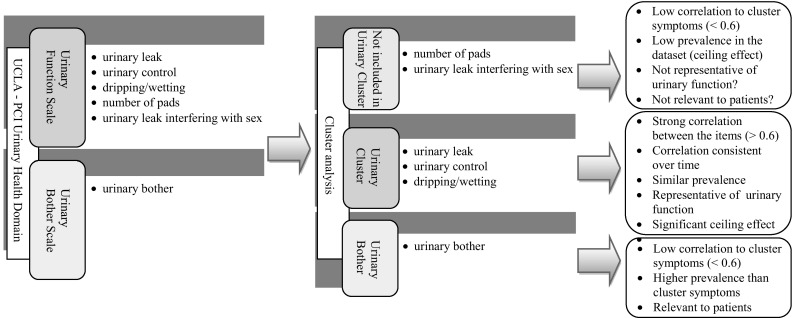
An illustration of two mechanisms of symptom grouping and how symptom clusters can contribute to the interpretation of patients reported outcome measures (PROMs) data. Using generic scales of the University of California, Los Angeles Prostate Cancer Index (UCLA-PCI) (*left*) and using cluster analysis to revise scale membership (*right*)

**Fig. 4:**
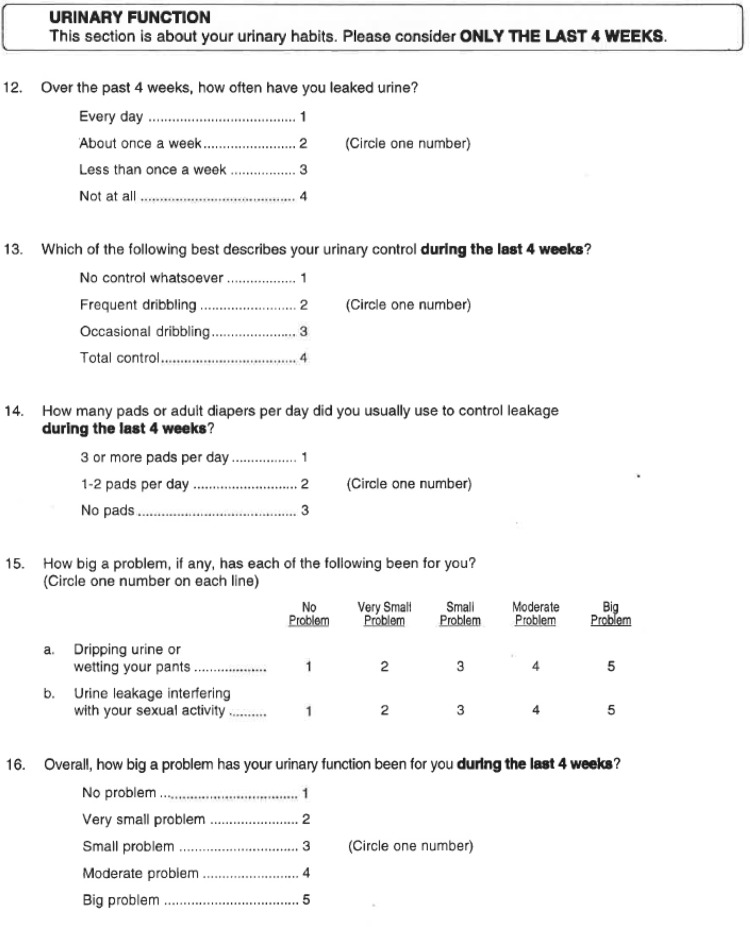
A section of the questionnaire used for recording scores for the Urinary Health Domain of the University of California, Los Angeles Prostate Cancer Index (UCLA-PCI) [6, 10]

Cluster analysis provide a study-specific approach to summarising PROMs data and to exploring trends for utilisation of PROMs in clinical practice [64]. This concept is illustrated in Fig. 3 using the UCLA-PCI Urinary Domain. The two mechanisms of symptoms grouping are illustrated (cluster analysis is used to revise scale membership). Symptom clusters contribute to the interpretation of PROMs data by grouping symptoms according to their prevalence and excluding items that are not representative of the same underlying health concern. “Urinary leak interfering with sex” has been shown not to be related to the other urinary function scale items. This item represents a link between sexual activity and urinary function. However, it did not cluster with the three urinary cluster symptoms. This may be due to the low prevalence of this item. Patients reported it rarely. Additionally, the analysis of missing values has shown that this item was left unanswered more often than any other urinary domain questions (Table 2). Both low prevalence and high degree of missingness, may reflect low levels of sexual activity within patients in the analysed study [14]. This item may be of lower relevance than other items and may not reflect urinary function. Therefore, including it in a summative score may lead to the loss of significant information.

Another implication for PROMs data analysis is observed for all the items in the urinary domain significant ceiling effect. This ceiling effect may be due to inadequate sensitivity of the scales adapted for each item. It may be that the scale levels (or ranges of scores) are not sensitive enough to capture the functional status of patients, and most patients score it at the upper limit of the range. Therefore, reporting the frequency of those items may not be insightful enough, and although, all the questions were standardised prior to analysis, a ceiling effect occurs for considerably more patients in the “number of pads” or “urinary leak interfering with sex” than for other urinary function items. However, the observed ceiling effect may also be due to the large numbers of patients not experiencing any dysfunction. Late effects from radiotherapy have been shown to occur in 5–10% of prostate cancer survivors [15, 35, 65, 66]. Nevertheless, the ceiling effect poses significant implications for data analysis and interpretation. Issues such as a low variance in the recorded data or data skewness due to abnormal distribution have to be accounted for and interpreted appropriately [67].

“Urinary bother” was the most prevalent item of the urinary domain. The baseline and acute scores of the “urinary bother” were lower than all the urinary function items. This has also been observed in other studies and patients reported lower bother scores than they rated their functional status [16, 68]. “Urinary bother” did not cluster with the urinary function items at any point in time and the correlation was low to moderate.

Urinary function scale contains five items that were developed to represent urinary function. However, two of the items have shown significantly lower prevalence as well as weak correlation compared to the remaining three items of the scale. Detailed correlation analysis has shown a consistently low correlation of “number of pads” and “urinary leak interfering with sex” with the other items. It is possible that they may not measure the same underling health issue or may not represent the urinary function. Therefore, including them in the summative score may lead to a loss of significant information due to the ceiling effect and weak relationship. This was reflected in the result of the cluster analysis. Cluster analysis could be applied to evaluate scale membership and to explore significant factors in PROMs data. Symptom clusters allow for study-specific approaches to be incorporated into PROMs data modelling techniques. The analysis can be used rather than grouping items with the original tool’s scales. Cronbach’s alpha analysis confirmed the clustering results. It has shown that by excluding those items from the urinary function scale, the reliability of the scale has not been compromised. The summative scores of the clusters also showed better predictive power than the summative scores obtained from the scales. The difference is not statistically significant but is consistent and is probably due to poorly correlated items included in the summative scores. Poor correlation, low variance as well as the degree of negative skewness should be investigated for each study when analysing PROMs. This may result in different groups of items or in the exclusion of some items from the summative scores. This may also advance PROMs data analysis and lead to extraction of more relevant information. It should be recognised that this study is based on an old tool (UCLA-PCI) that has largely been replaced with the EPIC [11]. However, because EPIC has been developed based on UCLA-PCI, the results of this study are still highly relevant. EPIC, similarly to UCLA-PCI (and other instruments used to investigate function and HRQOL in cancer patients), is a tool that uses multiple-item scales and requires calculation of composite scores to extract and analyse PROMs data.

### Conclusions

This study adds to knowledge on how cluster analysis can help to interpret and utilise PROMs. Scale membership was reviewed by cluster analysis, and the reliability of the Urinary Function Scale was evaluated. The approach of symptom clusters allows for a method of study-specific item grouping that can be used to calculate surrogate scores that simplify PROMs. Using summary scores of related items, rather than analysing symptoms in isolation, is essential because closely related variables exhibit a high degree of collinearity, which has implications for statistical approaches. This is also important in clinical decision making, and better health outcomes and HRQOL have been achieved when related symptoms were clinically considered and managed together [24, 25]. We recommend that when summarising and modelling PROMs data in clinical trials, scales membership should be reviewed. This is because the grouping of symptoms may vary for different studies, treatments and patient populations. Symptom clusters can be used for this purpose. They can be calculated individually for each study, and this can facilitate a study-specific rather than generic method of grouping items. This approach is more meaningful for the purpose of symptom management.

## Appendix

See Fig. 4.
